# Indiana Medical Journal, Evansville, Indiana

**Published:** 1854-07

**Authors:** 


*Indiana Medical Journal, Evansville, Indiana. Edited by* DRS. BYFORD AND RONALDS. *Quarterly.*

We have received the first number of the above work, which we welcome into the lists as a very neat and well conducted Journal.—An admirable feature in the No. before us is that it is made up of original communications almost entirely. This speaks most flatteringly for the zeal and acquirements of the medical men of that State, and the productions themselves tell well for the several contributors. Among others we notice those from the pen of Dr. Reid of Terre Haute, whose acquaintance we made and from whom we received many civilities five years since, when journeying to this country, all of which we have not forgotten. From the interest manifested by the contributors, we feel assured, as we sincerely hope, that it will be generously sustained.

